# Predictors of echocardiographic response in the Budapest-CRT upgrade trial

**DOI:** 10.1007/s00392-026-02963-x

**Published:** 2026-08-19

**Authors:** Eperke Merkel, Richárd Masszi, Bálint Lakatos, Robert Hatala, Boglárka Veres, Alexandra Fábián, István Osztheimer, László Gellér, Tamás Urbán, Csaba Horváth, Scott D. Solomon, Valentina Kutyifa, Attila Kovács, Béla Merkely, Annamária Kosztin

**Affiliations:** 1 Semmelweis University, Heart and Vascular Center, Budapest, Hungary; 2 Department of Cardiology and Angiology, National Institute of Cardiovascular Diseases and Slovak Medical University, Bratislava, Slovakia; 3 Cardiovascular Division, Brigham and Women’s Hospital, Harvard Medical School, Boston, Massachusetts USA; 4 Clinical Cardiovascular Research Center, University of Rochester, Rochester, NY USA

**Keywords:** CRT upgrade, Echocardiographic response, Heart failure

## Abstract

**Background and aims:**

Up to 30% of patients fail to respond after cardiac resynchronization therapy (CRT). While echocardiographic predictors of response are well described in de novo CRT, evidence in CRT upgrade populations remains limited. We investigated predictors of echocardiographic response in the BUDAPEST-CRT Upgrade trial cohort.

**Methods:**

In this randomized trial, 360 patients with heart failure and reduced ejection fraction (HFrEF) with priorly implanted pacemaker or ICD, ≥ 20% right ventricular (RV) pacing burden, and wide-paced QRS (≥ 150 ms) were randomized to CRT-D upgrade (*n* = 215) or ICD upgrade (*n* = 145). Echocardiographic response was defined as ≥ 15% reduction in left ventricular end-systolic volume (LVESV) at 12 months.

**Results:**

Among 275 patients with paired echocardiographic data, 161 (59%) were responders. Responders had fewer HF hospitalizations (0.6% vs. 7.0%, *p* = 0.003), more often received mineralocorticoid receptor antagonists (71% vs. 59%, *p* = 0.038), and showed a higher baseline RV pacing burden (88.9% vs. 83.4%, *p* = 0.025) than non-responders. Independent predictors of echocardiographic response were randomization to CRT-D (OR 5.57, 95% CI 2.56–12.12, *p* < 0.0001), greater RV pacing burden (OR 1.02 per %, 95% CI 1.00–1.04, *p* = 0.024), non-ischemic cardiomyopathy (OR 2.50, 95% CI 1.15–5.26, *p* = 0.021), and a lateral LV lead position (OR 8.79, 95% CI 1.28–60.39, *p* = 0.027).

**Conclusions:**

In patients with pacing-induced cardiomyopathy, CRT-D upgrade significantly improved echocardiographic outcomes. Non-ischemic etiology, higher RV pacing burden, and lateral LV lead positioning were associated with greater benefit. These results underscore the importance of tailored patient selection and lead placement strategies to optimize CRT-D upgrade outcomes.

**Graphical abstract:**

**Figure:**
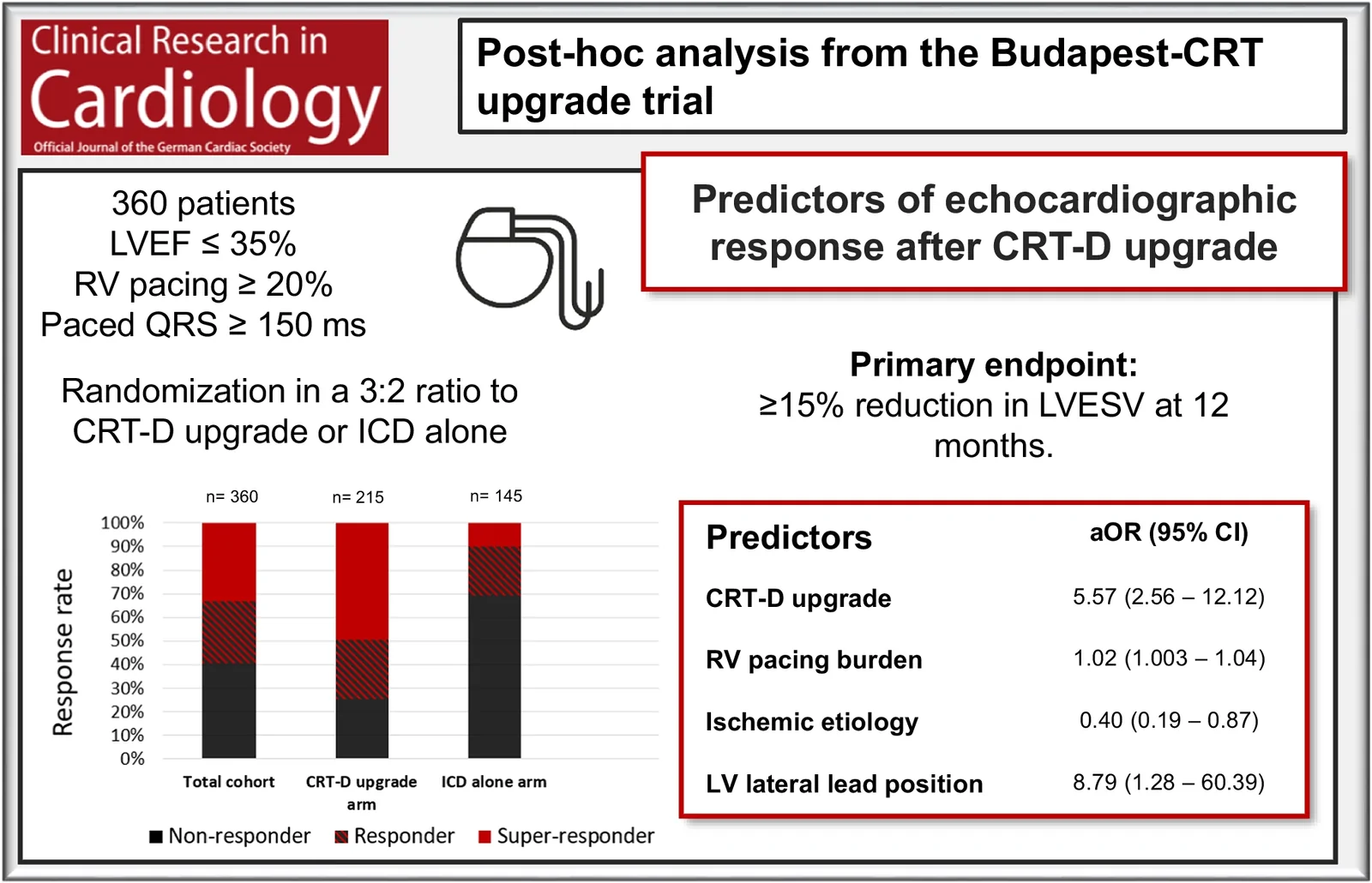

**Supplementary information:**

The online version contains supplementary material available at https://doi.org/10.1007/s00392-026-02963-x.

## Introduction

Pacing-induced cardiomyopathy occurs in a relevant proportion of patients; a clinically significant share of patients implanted with a pacemaker (PM) or implantable cardioverter defibrillator (ICD) will develop left ventricular systolic dysfunction due to the chronic right ventricular (RV) pacing [1, 2]. The BUDAPEST-CRT Upgrade trial proved enormous benefit of upgrading heart failure patients with a reduced ejection fraction (HFrEF) and a high RV pacing burden as compared to ICD alone. Upgrading to cardiac resynchronization therapy (CRT-D) substantially decreased the risk of the primary endpoint (all-cause mortality, heart failure (HF), or lack of reverse remodeling) and HF hospitalizations compared to ICD upgrade alone. This favorable effect was observed consistently in any subgroups indicating a robust and homogeneous treatment effect [3]. Also having a clear echocardiographic response with 10% relative increase on the CRT-D arm, while negligible change on the ICD arm during the course of the trial (3).

Despite the clear benefit in the echocardiographic response after de novo CRT implantation, a proportion of patients continue to show suboptimal response after CRT implantation, still highlighting the persistent challenge of non-response not only in de novo CRT but also after upgrade procedures. Predictors of response in de novo CRT trials have been studied extensively showing that female sex, non-ischemic cardiomyopathy, left bundle branch block (LBBB), and wide QRS interval [4–7] are all associated with a better long-term outcome. These factors, however, may not fully translate to the CRT upgrade population, where the underlying pacing-induced cardiomyopathy may play an additional role.

Therefore, the present substudy was undertaken to evaluate both clinical and procedural predictors of response in the BUDAPEST-CRT Upgrade trial cohort.

## Methods

### Study population

The rationale, design, and primary results of the BUDAPEST-CRT Upgrade trial have been published previously [3, 8, 9]. Briefly, 360 HFrEF patients with a previously implanted PM or ICD, chronic RV pacing burden (≥ 20%), and a wide-paced QRS (≥ 150 ms) were enrolled and randomized to CRT-D Upgrade (*n* = 215) or ICD upgrade alone (*n* = 145). Patients were treated with guideline-directed medical therapy (GDMT) and did not have a native intrinsic left bundle branch block.

The study protocol was approved by local and institutional ethics committees.

### Data and follow-up

Enrolled patients were followed for 12 months post-randomization. Outpatient follow-up visits were conducted at 1, 6, and 12 months, during which clinical parameters, ECG, device interrogation, echocardiographic assessments, and biochemical markers were collected. Additionally, the 6-min walk test (6-MWT) and EQ-5D-3L quality of life questionnaires were required at both baseline and the 12-month follow-up. In the procedural data, LV lead positions were assessed intraoperatively under fluoroscopy.

### Echocardiographic measurements

Echocardiographic data were sent to the Echocardiography Core Laboratory at Semmelweis University, Heart and Vascular Center (Budapest, Hungary) for a centralized evaluation. Digitally stored echocardiographic datasets were analyzed offline by two independent, experienced readers blinded to clinical information, randomization, and outcomes. Left ventricular volumes and left ventricular ejection fraction (LVEF) were measured using the biplane Simpson method at baseline (after randomization but before implantation) and again at the 12-month follow-up as recommended [10].

### Endpoints

Echocardiographic responders were classified by ventricular end-systolic volume (LVESV) reduction when it was greater than 15% at 12 months and additionally super-responder status was defined when LVESV reduction was greater than 30% [11]. Additionally, the BUDAPEST-CRT Upgrade trial’s primary composite endpoint (HF hospitalization, all-cause mortality, or less than 15% reduction in LVESV at 12 months) and the secondary endpoints (all-cause mortality or HF hospitalizations, LVESV change, LVEF change) were also assessed by the echocardiographic response, LV lead position and HF etiology.

Predictors of echocardiographic response as dichotomized variables were investigated as an intention-to-treat analysis except for the LV lead position. LV lead position was determined according to the short-axis location (anterior, lateral, or posterior), documented at the discretion of the implanting physician, and analyzed in patients randomized to the CRT-D arm as well as in those who crossed over from the ICD arm. Patients with an anterior position were used as a control group.

### Statistical analysis

Continuous variables are expressed as mean ± standard deviation, or as medians with interquartile range (25th–75th percentile), as appropriate. Categorical variables are summarized with frequencies and percentages. Baseline clinical characteristics were compared based on responder status using a *t*-test for normally distributed continuous variables, the Mann–Whitney *U* test for non-normally distributed variables, while *χ*^2^ test was used for dichotomous variables as appropriate.

The primary outcome was analyzed using logistic regression due to its binary component, and the effect size was expressed as adjusted and unadjusted odds ratios (OR) with associated 95% confidence intervals (CI). Time-to-event secondary outcomes (composite endpoint of all-cause mortality and HF hospitalization and all-cause mortality alone) were analyzed by Cox proportional hazards models. HF hospitalization alone as an outcome was specified in a post hoc manner. Changes in LVESV, LVEDV, LVEF, and NT-proBNP were analyzed by linear regression.

The primary outcome analysis was done in the modified intention-to-treat (ITT) population: patients with missing results of the echocardiography component of the primary outcome and who did not meet the primary outcome through the other components (HF or death) were excluded. However, they were included in all other analyses based on ITT principles. Per-protocol analyses were also conducted for echocardiographic response and LV lead positions. The prespecified adjustment factors were age, sex, country, ischemic etiology, diabetes mellitus, secondary prevention ICD, and baseline New York Heart Association (NYHA) class, although country and baseline NYHA class were not applicable due to the small sample size in the subgroups. Estimation of the treatment effect itself in the subgroups was analyzed by the application of the above-mentioned modeling strategy to each subgroup. Interaction *p*-values were also calculated to test whether the effects significantly differed between the subgroups. The presented *p*-values and the width of the CIs were not adjusted for multiplicity. Statistical analyses were performed by using Stata version 18.0 (StataCorp., College Station, TX, USA). This trial is registered at ClinicalTrials.gov, NCT02270840.

## Results

### Baseline clinical characteristics

The baseline clinical characteristics have been published previously [8]. Baseline and 12-month echocardiographic data were available for 275 (76%) patients, 161 (59%) patients proved to be responders, and 114 (41%) non-responders (Table 1). One hundred twenty-six patients (78%) of the responder patients were from the CRT-D arm. Within the responder group, 92 (34%) of the patients proved to be super-responders. On the CRT-D upgrade arm, 41 (25%) patients were non-responders and 126 (75%) patients were responders; of these patients, 81 (64%) proved to be super-responders. Within the CRT-D upgrade arm, biventricular pacing rate did not differ between responders and non-responders (98% [95–100%] and 99% [97–99.9%], *p* = 0.89, respectively). On the ICD alone arm, 73 (68%) patients were non-responders, and 35 (32%) patients were responders, of whom one-third were cross-overs to the CRT-D upgrade arm (10 patients). On the ICD alone arm, within the responder group, 11 patients (10%) experienced an LVESV reduction greater than 30%.

**Table 1:** Baseline clinical characteristics of patients by echocardiographic response

Clinical characteristics	Total	Non-responder	Responder	*p*-value
	*N* = 275	*N* = 114	*N* = 161	
Age, years (SD)	72.8 (7.9)	72.4 (8.3)	73.1 (7.6)	0.48
Sex (male), *n* (%)	243 (88.4%)	105 (92.1%)	138 (85.7%)	0.10
BMI, kg/m^2^ (SD)	28.6 (4.6)	28.7(4.7)	28.6 (4.56)	0.91
AMI, *n* (%)	133 (48.4%)	63 (55.3%)	70 (43.5%)	0.054
CABG, *n* (%)	72 (26.2%)	32 (28.1%)	40 (24.8%)	0.55
PCI, *n* (%)	107 (38.9%)	52 (45.6%)	55 (34.2%)	0.055
Diabetes, *n* (%)	105 (38.2%)	46 (40.4%)	59 (36.6%)	0.53
Hyperlipidemia, *n* (%)	131 (47.6%)	62 (54.4%)	69 (42.9%)	0.059
Hypertension, *n* (%)	219 (79.6%)	87 (76.3%)	132 (82.0%)	0.25
Prior VT/VF, *n* (%)	64 (23.3%)	33 (28.9%)	31 (19.3%)	0.061
Atrial fibrillation	156 (56.7%)	67 (58.8%)	89 (55.3%)	0.56
HF hospitalization 12 months prior to enrollment, *n* (%)	137 (49.8%)	61 (53.5%)	76 (47.2%)	0.30
Prior pacemaker type				0.31
CRT with plug, *n* (%)	1 (0.4%)	1 (0.9%)	0 (0.0%)	
ICD, *n* (%)	81 (29.5%)	37 (32.5%)	44 (27.3%)	
PM, *n* (%)	193 (70.2%)	76 (66.7%)	117 (72.7%)	
LVEF, % (SD)	25.2 (6.7)	25.6 (6.7)	25.0 (6.6)	0.44
LVESV, mL (SD)	172.9 (65.6)	173.6 (69.5)	172.5 (63.0)	0.89
LVEDV, mL (SD)	229.2 (78.1)	231.3 (83.2)	227.8 (74.5)	0.71
NT-pro-BNP, pg/mL (IQR)	2128 (1254–3978)	2041 (1433–3743)	2255 (1218–4096)	0.87
eGFR, mL/min/1.73 m (SD)	59.3 (18.9)	59.7 (18.0)	59.0 (19.6)	0.78
Baseline NYHA class				0.69
NYHA II, *n* (%)	139 (50.5%)	56 (49.1%)	83 (51.6%)	
NYHA III-IV, *n* (%)	136 (49.5%)	58 (50.9%)	78 (48.4%)	
Baseline 6-MWT, m (SD)	285.6 (114.6)	285.3 (128.5)	285.7 (104.2)	0.98
Baseline EQ-5D-3L, score (SD)	0.688 (0.267)	0.689 (0.261)	0.688 (0.271)	0.98
ACE inhibitors, *n* (%)	203 (73.8%)	82 (71.9%)	121 (75.2%)	0.55
ARB, *n* (%)	50 (18.2%)	22 (19.3%)	28 (17.4%)	0.69
β-Blockers, *n* (%)	255 (92.7%)	105 (92.1%)	150 (93.2%)	0.74
Ca^2^⁺ channel blockers, *n* (%)	29 (10.5%)	5 (4.4%)	24 (14.9%)	0.005
Loop diuretics, *n* (%)	222 (80.7%)	96 (84.2%)	126 (78.3%)	0.22
Amiodarone, *n* (%)	67 (24.4%)	29 (25.4%)	38 (23.6%)	0.73
MRA, *n* (%)	181 (65.8%)	67 (58.8%)	114 (70.8%)	0.038
Percent RV pacing prior to enrollment, % (SD)	86.6 (20.0)	83.4 (21.6)	88.9(18.5)	0.025

Responders and non-responders were of comparable age (73.1 ± 7.6 years vs. 72.4 ± 8.3 years, *p* = 0.48), sex (male 85.7% vs. 92.1%, *p* = 0.10), HF symptoms at baseline (NYHA II 49% vs. 51%; NYHA III–IV 51% vs. 49%; *p* = 0.69), and comorbidity burden, as diabetes (DM) and hypertension (HT) occurred at a similar rate in both groups (DM 36% vs. 40%; *p* = 0.53 and HT 82% vs. 76%; *p* = 0.25). Responders were more frequently treated with mineralocorticoid receptor antagonists (71% vs. 59%; *p* = 0.038) and had a higher RV pacing burden at enrollment (88.9% vs. 83.4%; *p* = 0.025) (Table 1).

Within the responder patient group, super-responders showed a tendency to be older compared to patients experiencing an echocardiographic response greater than 15% but less than 30% [71.7 (8.2) vs. 74.1 (6.9) years of age; *p* = 0.054], respectively. Similarly, a trend could be observed regarding sex, as male sex was less frequent among super-responders (81.5% in super-responders vs. 91.3%; *p* = 0.079). Super-responders were less likely to have prior myocardial infarction (32.6% vs. 58.0%; *p* = 0.001) and percutaneous coronary intervention (21.7% vs. 50.7%; *p* < 0.001). Super-responders were implanted with a pacemaker rather than an ICD compared to responders (super-responders PM 63.8%, ICD 20.7% vs. responders PM 79.3%, ICD 20.7%; *p* = 0.028) (Supplementary Table 1).

### Predictors of response

During the mean follow-up time of 12.4 months, responder status was associated with being on the CRT-D arm (aOR 5.57; 95% CI 2.56 to 12.12; *p* < 0.001), higher RV pacing burden (aOR 1.02; 95% CI 1.003 to 1.04; *p* = 0.024), ischemic etiology (aOR 0.40; 95% CI 0.19 to 0.87; *p* = 0.021), and LV lead position (lateral aOR 8.79; 95% CI 1.28 to 60.39; *p* = 0.027). Patients showing echocardiographic response were less likely to experience multiple HF hospitalizations before enrollment (0.6% vs. 7%; *p* = 0.003) (Table 2), despite the comparable baseline echocardiographic LV dimensions. Based on the per-protocol principle, similarly, responder status was associated with CRT-D upgrade (aOR 5.39; 95% CI 2.35 to 12.37; *p* < 0.001), higher RV pacing burden (aOR 1.02; 95% CI 1.003 to 1.04; *p* = 0.018), ischemic etiology (aOR 0.39; 95% CI 0.18 to 0.84; *p* = 0.017), and LV lead position (lateral aOR 7.65; 95% CI 1.12 to 52.51; *p* = 0.038).

**Table 2:** Predictors of echocardiographic response by multivariate logistic regression

Responder status	Odds ratio	95% CI	*p*-value
CRT-D arm	5.57	2.56 to 12.12	** < 0.0001**
Age	1.03	0.98 to 1.07	0.27
Sex	0.72	0.24 to 2.20	0.56
Baseline NYHA III	0.84	0.39 to 1.82	0.67
Baseline NYHA IV	0.20	0.02 to 2.56	0.21
BMI	1.02	0.93 to 1.11	0.72
Diabetes mellitus	0.68	0.30 to 1.52	0.21
RV pace (%)	1.02	1.003 to 1.04	**0.024**
Atrial fibrillation	1.80	0.77 to 4.21	0.17
Ischemic etiology	0.40	0.19 to 0.87	**0.021**
Creatinine	1.01	0.99 to 1.02	0.311
LVEF	1.00	0.93 to 1.07	0.99
LV lateral position	8.79	1.28 to 60.39	**0.027**
LV posterior position	2.77	0.36 to 21.12	0.33

the bold values ment reaching the significance level of 0.05

Within the responder patient groups, super-response was associated with being on the CRT-D upgrade arm (OR 6.77; 95% CI 1.91 to 24.01; *p* = 0.003), and ischemic etiology decreased its odds (OR 0.17; 95% CI 0.06 to 0.51; *p* = 0.002) (Supplementary Table 2).

### LV electrode position

In this subgroup analysis, we studied only those patients who were randomly assigned to the CRT-D arm (*n* = 215) and those who were cross-overs from the ICD arm (*n* = 27). LV lead location was available for 223 (89%) patients with a median follow-up time of 12.0 months. In 173 (78%) patients, LV lead was implanted into a lateral position, 36 (16%) in a posterior, and 14 (6%) in an anterior position.

By the LV lead positions, there was no difference between the primary outcome (lateral vs. anterior OR 0.31; 95% CI 0.08 to 1.20, *p* = 0.09, posterior vs. anterior OR 0.58; 95% CI 0.14 to 2.50, *p* = 0.47) (Table 3).

**Table 3:** Outcomes by LV lead short-axis position

Endpoints	Measure of effect	Unadjusted odds ratio or difference (95% CI)	*p*-value	Adjusted odds ratio or difference (95% CI)	*p*-value
Primary outcome					
Anterior	REFERENCE				
Lateral	OR	0.33 (0.09 to 1.22)	0.097	0.31 (0.08 to 1.26)	0.10
Posterior	OR	0.58 (0.14 to 2.50)	0.47	0.56 (0.12 to 2.70)	0.47
LVEF change from baseline to 12 months—%					
Lateral vs. anterior	Difference	+ 7.51 (1.34 to 13.67)	0.017	+ 7.43 (1.25 to 13.62)	0.019
Lateral vs. posterior	Difference	+ 4.64 (0.97 to 8.31)	0.014	+ 4.46 (0.78 to 8.15)	0.02
Posterior vs. anterior	Difference	− 0.06 (− 0.24 to 0.12)	0.49	+ 3.0 (− 3.92 to 9.93)	0.39
LVESV change from baseline to 12 months—mL					
Lateral vs. anterior	Difference	− 0.16 (− 0.32 to 0.001)	0.054	− 0.17 (− 0.32 to − 0.01)	0.03
Lateral vs. posterior	Difference	− 0.10 (− 0.19 to 0.001)	0.051	− 0.08 (− 0.17 to 0.01)	0.10
Posterior vs. anterior	Difference	− 0.06 (− 0.24 to 0.12)	0.49	− 0.09 (− 0.26 to 0.08)	0.31

**Fig. 1:**
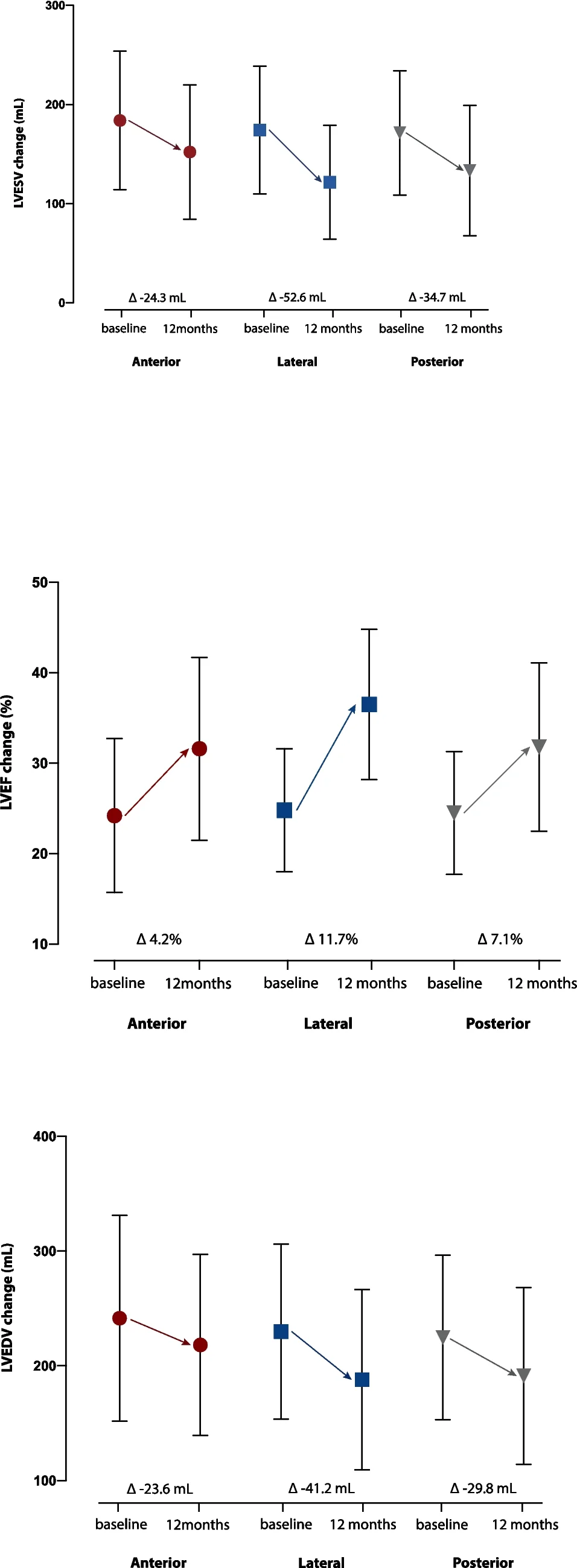
Echocardiographic response by LV lead short-axis position (LVEF, LVESV, and LVEDV)

Positive LVEF change was more pronounced in those with lateral LV lead location compared with anterior or posterior positions (adjusted LVEF difference lateral vs. anterior 7.43%, 95% CI 1.25 to 13.62; *p* = 0.02) (adjusted LVEF difference lateral vs. posterior 4.46%, 95% CI 0.78 to 8.15; *p* = 0.02), while those with posterior leads did not differ from the anterior ones (adjusted LVEF difference 3.00%, 95% CI − 3.92 to 9.93; *p* = 0.39). Decrease in ESV was also more notable with a lateral position as compared to the other locations (lateral vs. anterior adjusted ESV difference − 0.17 mL, 95% CI − 0.32 to − 0.01; *p* = 0.03, posterior vs. anterior adjusted ESV difference − 0.09 mL, 95% CI − 0.26 to 0.08; *p* = 0.31, posterior vs. lateral adjusted ESV difference − 0.08 mL, 95% CI − 0.17 to 0.01; *p* = 0.10) (Fig. 1).

### Heart failure etiology

At enrollment, 209 (58%) patients had ischemic etiology, of whose risk to the primary endpoint was 78% higher as compared to non-ischemic patients (aOR 1.78; 95% CI 1.02 to 3.11; *p* = 0.042); however, they did not show a higher risk for all-cause mortality or HFH (aOR 0.84; 95% CI 0.47 to 1.48; *p* = 0.54) during the median follow-up time of 12 months. Having ischemic etiology also decreased the odds of showing echocardiographic response (aOR 0.40; 95% CI 0.19 to 0.87; *p* = 0.021).

**Fig. 2:**
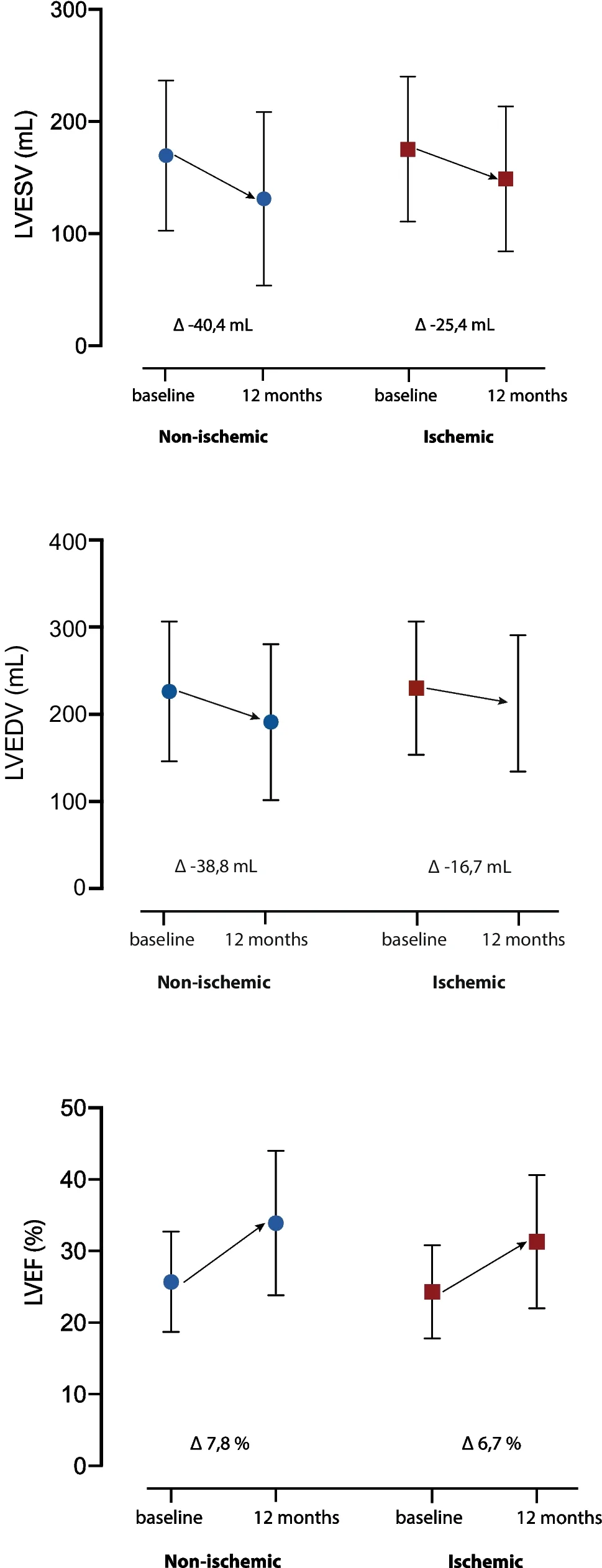
Echocardiographic response by heart failure etiology (LVEF, LVESV, and LVEDV)

Despite these results, the effect of CRT-D upgrade on the primary endpoint was similar in patients with ischemic and non-ischemic etiology (ischemic aOR 0.11; 95% CI 0.05 to 0.23; *p* < 0.001; non-ischemic aOR 0.12; 95% CI 0.05 to 0.28; *p* < 0.001; interaction *p* = 0.91). Also, CRT-D upgrade improved the echocardiographic response, with a decrease in LVEDV and LVESV, and a significant interaction between treatment effect and HF etiology [(ischemic adjusted LVEDV difference − 25.24 mL, 95% CI − 40.32 to − 10.17, *p* = 0.001; non-ischemic − 55.79 mL, 95% CI − 77.96 to − 33.62, *p* < 0.001; interaction *p* = 0.013) and LVESV difference interaction *p* = 0.025)] (Table 4 and Fig. 2).

**Table 4:** Outcomes by treatment arm in patients with ischemic heart disease

Endpoints	Measure of effect	Unadjusted odds ratio, hazard ratio or difference (95% CI)	*p*-value	Adjusted odds ratio, hazard ratio or difference (95% CI)	*p*-value
Primary outcome					
Composite endpoint of all-cause death or heart failure hospitalization or < 15% end-systolic volume decrease	OR	0.12 (0.06 to 0.25)	< 0.0001	0.11 (0.05 to 0.23)	< 0.0001
Secondary outcomes					
Composite endpoint of all-cause death or HF hospitalizations	HR	0.30 (0.16 to 0.57)	< 0.0001	0.32 (0.16 to 0.63)	0.001
HF hospitalization	HR	0.25 (0.12 to 0.52)	< 0.0001	0.25 (0.12 to 0.54)	< 0.0001
LVEF change from baseline to 12 months—%	Difference	+ 9.07 (6.50 to 11.65)	< 0.0001	+ 9.01 (6.32 to 11.70)	< 0.0001
LVESV change from baseline to 12 months—mL	Difference	− 38.68 (− 50.95 to − 26.40)	< 0.0001	− 38.01 (− 50.74 to − 25.29)	< 0.0001
LVEDV change from baseline to 12 months—mL	Difference	− 26.49 (− 40.88 to − 12.09)	< 0.0001	− 25.24 (− 40.32 to − 10.17)	0.001

## Discussion

In this substudy analysis of the BUDAPEST-CRT Upgrade trial cohort, we demonstrated a significant benefit of CRT-D upgrade on the echocardiographic response at 12 months as compared to ICD alone. The proportion of patients who reached echocardiographic reverse remodeling was high; furthermore, over one-third of the patients proved to be super-responders. Non-ischemic etiology, high RV pacing burden at enrollment, and having an LV lead in a lateral position were identified as independent predictors of echocardiographic response.

Non-response to therapy is still recognized as a significant concern, as the rate of non-response reaches 30–40% after de novo CRT implantation, understudied in patients after CRT upgrade. Previously, several parameters at patient selection, implantation, or during follow-up were proved to be predictors of echocardiographic response in de novo CRT patients as a surrogate of long-term outcome: female sex, non-ischemic etiology, and LV lead position are the most important ones.

In this cohort, more than two-thirds of the patients responded optimally; moreover, a high proportion of patients showed super-response, a much higher rate than seen in de novo CRT trials. When the optimal patient selection by the baseline characteristics was investigated, we found that both responders and non-responders were an advanced-stage heart failure group with no striking differences, but in ischemic etiology and RV pacing burden.

When predictive factors were assessed, the above-mentioned factors still remain relevant in this cohort. Heart failure etiology emerged as a potential predictor of response in our analysis, as previously observed in de novo CRT studies. Substudies of these milestone trials proved the efficacy of CRT in both ischemic and non-ischemic cardiomyopathy [6, 12, 13]. Nonetheless, ischemic etiology is associated with an elevated risk of all-cause mortality [14]. Patients undergoing CRT show echocardiographic response and reverse remodeling regardless of etiology, though the extent of improvement is less pronounced in ischemic etiology [6, 12, 15, 16]. In the BUDAPEST-CRT Upgrade trial, comparable results were observed, even after the elimination of the supposed mechanism leading to the deterioration of systolic function; patients with ischemic etiology showed less favorable echocardiographic response.

RV pacing rate is a relevant predictor in those undergoing CRT upgrade procedures; conflicting data emerged on its significance from retrospective and observational data [17, 18]. Chronic RV pacing’s deleterious effect has been observed extensively, first an RV pace greater than 40% was associated with an elevated risk of heart failure, later a lower cut-off value of 20% was identified as potentially harmful [1, 2]. In our cohort, the mean RV pacing rate at enrollment exceeded 85% and for every percentage increase in RV pacing, the odds of echocardiographic response increased by 2%, supporting the fact that pacing-induced dyssynchrony is highly reversible with CRT upgrade.

Therefore, a novel and clinically important observation in our study is the substantially higher proportion of super-responders (≥ 30% LVESV reduction) compared with landmark de novo CRT trials, including MADIT-CRT and CARE-HF (48% vs. ~ 30%). However, it should be noted that these trials did not exclusively enroll patients with LBBB; the inclusion criteria were based on QRS duration [15, 19]. This likely reflects the distinct pathophysiology of pacing-induced dyssynchrony, where the structural and electrical remodeling may be more reversible compared to intrinsic LBBB. In these patients, CRT upgrade not only restores synchrony but also drives remarkable improvements in systolic function and reverse remodeling, possibly explaining the superior response.

During implantation, positioning the LV lead at the site of the latest activated area of the LV resulted in better echocardiographic response and outcomes, especially in patients with typical LBBB [20, 21]. A MADIT-CRT substudy found a significant benefit of lateral and posterior lead positions on the risk of ventricular arrhythmias, all-cause mortality, and HF events at long-term, but apical locations were associated with poorer clinical outcomes [22–24]. Observational data found superiority of lateral positions with long-term mortality over both anterior and posterior positions [21]. Our findings align with data on de novo CRT, as in this substudy, we found that CRT-D upgrade was beneficial regardless of LV lead position; however, there was a greater echocardiographic response in patients with lateral LV lead positions compared to posterior or anterior positions. The similar benefits are most likely attributed to the comparable electromechanical dyssynchrony caused by RV pacing and intrinsic LBBB seen in some HFrEF patients [25].

## Conclusion

Cardiac resynchronization therapy upgrade significantly improves echocardiographic outcomes in patients with pacing-induced cardiomyopathy. Our findings demonstrate that non-ischemic etiology, lateral LV lead positioning, and a higher RV pacing burden are strong, independent predictors of response. These results emphasize the importance of early patientreferral andoptimal LV lead placement to maximize the benefits of CRT upgrade. By identifying patients most likely to respond, this study provides evidence to support a personalized, precision-based approach to device therapy.

## Limitations

Some limitations of this substudy should be noted. The specific inclusion and exclusion criteria may influence the results, the generalizability, and the number of subjects in different subgroups is limited. The underrepresentation of women in the trial may have limited the ability to evaluate sex as an independent predictor of response and clinical outcomes. Echocardiographic response could not be evaluated when data was missing at any timepoint. Long-axis LV lead positions were not documented, and short-axis positions were assessed by the implanting physician. Although echocardiographic response serves as a valuable surrogate endpoint after CRT implantation, it does not always translate into a corresponding clinical improvement. Changes in GDMT were not documented which could potentially influence our results.

## Supplementary information

Below is the link to the electronic supplementary material.

ESM 1 (DOCX 25.9 KB)

## Data Availability

The data underlying this article will be shared on reasonable request to the corresponding author.
