# Supplementary material for: Predictors of echocardiographic response in the Budapest-CRT upgrade trial
Source: Clin Res Cardiol. 2026 Aug 19;115(12):2206–15. doi: 10.1007/s00392-026-02963-x (PMC13751761; doi:10.1007/s00392-026-02963-x)
Supplement: Supplementary file 1 [file 392_2026_2963_MOESM1_ESM.docx]

| **Clinical characteristics** | **Total** | **Responder** | **Super-responder** | **p-value** |
| --- | --- | --- | --- | --- |
|  | N=161 | N=69 | N= 92 |  |
| Age, years (SD) | 73.1 (7.6) | 71.7 (8.2) | 74.1 (6.9) | 0.054 |
| Sex (Male), n (%) | 138 (85.7%) | 63 (91.3%) | 75 (81.5%) | 0.079 |
| BMI, kg/m2 (SD) | 28.6 (4.56) | 28.1 (4.5) | 29.0 (4.6) | 0.25 |
| AMI, n (%) | 70 (43.5%) | 40 (58.0%) | 30 (32.6%) | **0.001** |
| CABG, n (%) | 40 (24.8%) | 21 (30.4%) | 19 (20.6%) | 0.16 |
| PCI, n (%) | 55 (34.2%) | 35 (50.7%) | 20 (21.7%) | **<0.001** |
| Diabetes, n (%) | 59 (36.6%) | 27 (39.1%) | 32 (34.8%) | 0.57 |
| Hyperlipidemia, n (%) | 69 (42.9%) | 16 (37.7%) | 43 (46.7%) | 0.25 |
| Hypertension, n (%) | 132 (82.0%) | 50 (72.5%) | 82 (89.1%) | **0.006** |
| Prior VT/VF, n (%) | 31 (19.3%) | 17 (24.6%) | 14 (15.2%) | 0.13 |
| Atrial fibrillation | 89 (55.3%) | 37 (53.6%) | 33 (35.9%) | 0.71 |
| HF hospitalization 12 months prior to enrollment, n (%) | 76 (47.2%) | 31 (44.9%) | 45 (48.9%) | 0.62 |
| Prior pacemaker type |  |  |  | **0.028** |
| ICD, n (%) | 44 (27.3%) | 25 (36.2%) | 19 (20.7%) |  |
| PM, n (%) | 117 (72.7%) | 44 (63.8%) | 73 (79.3%) |  |
| LVEF, % (SD) | 25.0 (6.6) | 24.2 (5.8) | 25.4 (7.2) | 0.31 |
| LVESV, mL (SD) | 172.5 (63.0) | 173.8 (68.5) | 171.5 (58.9) | 0.81 |
| LVEDV, mL (SD) | 227.8 (74.5) | 227.8 (80.7) | 227.9 (69.8) | 227.9 (69.8) |
| NT-pro-BNP, pg/ml (IQR) | 2255  (1218-4096) | 2229  (1202-3818) | 2268  (1218-4309) | 0.66 |
| eGFR, mL/min/1.73 m (SD) | 59.0 (19.6) | 59.2 (19.6) | 58.9 (19.7) | 0.92 |
| Baseline NYHA class |  |  |  | 0.86 |
| NYHA II, n (%) | 83 (51.6%) | 35 (50.7%) | 48 (52.2%) |  |
| NYHA III-IV, n (%) | 78 (48.4%) | 34 (49.3%) | 44 (47.8%) |  |
| Baseline 6-MWT, m (SD) | 285.7 (104.2) | 296.2 (116.4) | 277.9 (94.2) | 0.30 |
| Baseline EQ-5D-3L, score (SD) | 0.688 (0.271) | 0.692 (0.183) | 0.684 (0.265) | 0.87 |
| ACE Inhibitors, n (%) | 121 (75.2%) | 54 (78.3%) | 67 (72.8%) | 0.43 |
| ARB, n (%) | 28 (17.4%) | 12 (17.4%) | 16 (17.4%) | 1.00 |
| β-Blockers, n (%) | 150 (93.2%) | 64 (92.8%) | 86 (93.5%) | 0.86 |
| Ca²⁺ Channel Blockers, n (%) | 24 (14.9%) | 8 (11.6%) | 16 (17.4%) | 0.31 |
| Loop Diuretics, n (%) | 126 (78.3%) | 54 (78.3%) | 72 (78.3%) | 1.00 |
| Amiodarone, n (%) | 38 (23.6%) | 20 (29.0%) | 18 (19.6%) | 0.16 |
| MRA, n (%) | 114 (70.8%) | 48 (69.6%) | 66 (71.7%) | 0.76 |
| Percent RV pacing prior to enrollment, % (SD) | 88.9 (18.5) | 87.8 (19.5) | 89.7 (17.8) | 0.60 |

Table 1. Baseline clinical characteristics of patients within the responder group

| **Responder status** | **Odds ratio** | **95% CI** | **p-value** |
| --- | --- | --- | --- |
| CRT-D arm | 6.77 | 1.91 to 24.01 | **0.003** |
| Age | 1.07 | 1.00 to 1.15 | 0.059 |
| Sex | 0.68 | 0.17 to 2.72 | 0.58 |
| Baseline NYHA III | 1.06 | 0.39 to 2.85 | 0.91 |
| BMI | 1.09 | 0.97 to 1.23 | 0.15 |
| Diabetes mellitus | 0.74 | 0.27 to 2.00 | 0.55 |
| RV pace (%) | 1.02 | 0.98 to 1.03 | 0.68 |
| Atrial fibrillation | 1.02 | 0.73 to 2.78 | 0.97 |
| Ischemic etiology | 0.17 | 0.06 to 0.51 | **0.002** |
| Creatinine | 1.00 | 0.98 to 1.01 | 0.63 |
| LVEF | 1.01 | 0.91 to 1.12 | 0.81 |
| LV lateral position | 0.53 | 0.13 to 2.11 | 0.37 |

Table 2. Predictors of echocardiographic super-response by multivariate logistic regression
